# A novel software program for detection of potential air emboli during cardiac surgery

**DOI:** 10.1186/1476-7120-13-3

**Published:** 2015-01-12

**Authors:** Frank Secretain, Andrew Pollard, Mesbah Uddin, Christopher G Ball, Andrew Hamilton, Robert C Tanzola, Joelle B Thorpe, Brian Milne

**Affiliations:** Department of Mechanical and Materials Engineering, Queen’s University, Kingston, ON K7L 3N6 Canada; Department of Mathematics and Statistics, Queen’s University, Kingston, ON Canada; Department of Mechanical Engineering, University of North Carolina, Charlotte, NC USA; Department of Pathology and Laboratory Medicine, University of Ottawa, Ottawa, ON Canada; Department of Surgery, Queen’s University, Kingston, ON Canada; Department of Anesthesiology & Perioperative Medicine, Queen’s University, Kingston, ON Canada

**Keywords:** Cardiac surgery, Cardiopulmonary bypass, Air emboli, Cerebral emboli, Neurological dysfunction, Myocardial dysfunction, Ultrasound, Transcranial doppler, Transesophageal echocardiography

## Abstract

**Background:**

Risks associated with air emboli introduced during cardiac surgery have been highlighted by reports of postoperative neuropsychological dysfunction, myocardial dysfunction, and mortality. Presently, there are no standard effective methods for quantifying potential emboli in the bloodstream during cardiac surgery. Our objective was to develop software that can automatically detect and quantify air bubbles within the ascending aorta and/or cardiac chambers during cardiac surgery in real time.

**Findings:**

We created a software algorithm (“Detection of Emboli using Transesophageal Echocardiography for Counting, Total volume, and Size estimation”, or DETECTS™) to identify and measure potential emboli present during cardiac surgery using two-dimensional ultrasound. An *in vitro* experiment was used to validate the accuracy of DETECTS™ at identifying and measuring air emboli. An experimental rig was built to correlate the ultrasound images to high definition camera images of air bubbles created in water by an automatic bubbler system. There was a correlation between true bubble size and the size reported by DETECTS™ in our *in vitro* experiment (r = 0.76). We also tested DETECTS™ using TEE images obtained during cardiac surgery, and provide visualization of the software interface.

**Conclusions:**

While monitoring the heart during cardiac surgery using existing ultrasound technology and DETECTS™, the operative team can obtain real-time data on the number and volume of potential air emboli. This system will potentially allow de-airing techniques to be evaluated and improved upon. This could lead to reduced air in the cardiac chambers after cardiopulmonary bypass, possibly reducing the risk of neurological dysfunction following cardiac surgery.

**Electronic supplementary material:**

The online version of this article (doi:10.1186/1476-7120-13-3) contains supplementary material, which is available to authorized users.

## Introduction

During cardiac surgery, particularly during cardiopulmonary bypass (CPB), air may be introduced into circulation [1, 2]. These air bubbles can cause neurological dysfunction, myocardial dysfunction, and even death [2–6]. To avoid these potentially fatal effects, all surgeons employ a variety of de-airing techniques including venting and altering the patient’s position, and carbon dioxide field flooding in an attempt to reduce the impact of air emboli [7]. However, these de-airing techniques are largely non-standardized. Additionally, the current methods for identifying potential air emboli do not allow for rapid, automatic, real-time detection during cardiac surgery. Having this capability would better enable surgeons to assess their de-airing techniques and adjust where necessary.

Transcranial Doppler (TCD) focused on the middle cerebral artery may assist in the identification of cerebral microemboli [8, 9]; however, this method does not detect the emboli until they reach the brain, where they pose a serious neurological threat. Thus, TCD cannot be used as a preventative solution. Transesophageal echocardiography (TEE) can be used to visualize potential emboli in the heart and circulation once the patient has been separated from CPB. TEE requires trained personnel to identify potential emboli, and does not provide a size estimate for each embolus [10]. The ability to automatically detect the presence and estimate the size of potential air emboli in the heart in real time would allow the operating team to evaluate their de-airing techniques, and might contribute to the improvement and development of new and more effective de-airing practices. In turn, this may lead to a reduction in air emboli, thereby decreasing the rates of neurological dysfunction after cardiac surgery using CPB.

Here, we present a new software algorithm named Detection of Emboli using Transesophageal Echocardiography for Counting, Total volume and Size estimation (DETECTS™) as a solution to identify potential emboli in a rapid, real-time, and automatic manner. This program was designed to operate as an online system to benefit the operative team during cardiac surgery. We also present the results of an *in vitro* experiment validating the accuracy with which DETECTS™ quantifies the number and size of potential air emboli. Use of DETECTS™ in cardiac surgery patients was approved by the Queen’s University Health Sciences & Affiliated Teaching Hospitals Research Ethics Board.

### DETECTS™ software algorithm and interface

**Figure 1:**
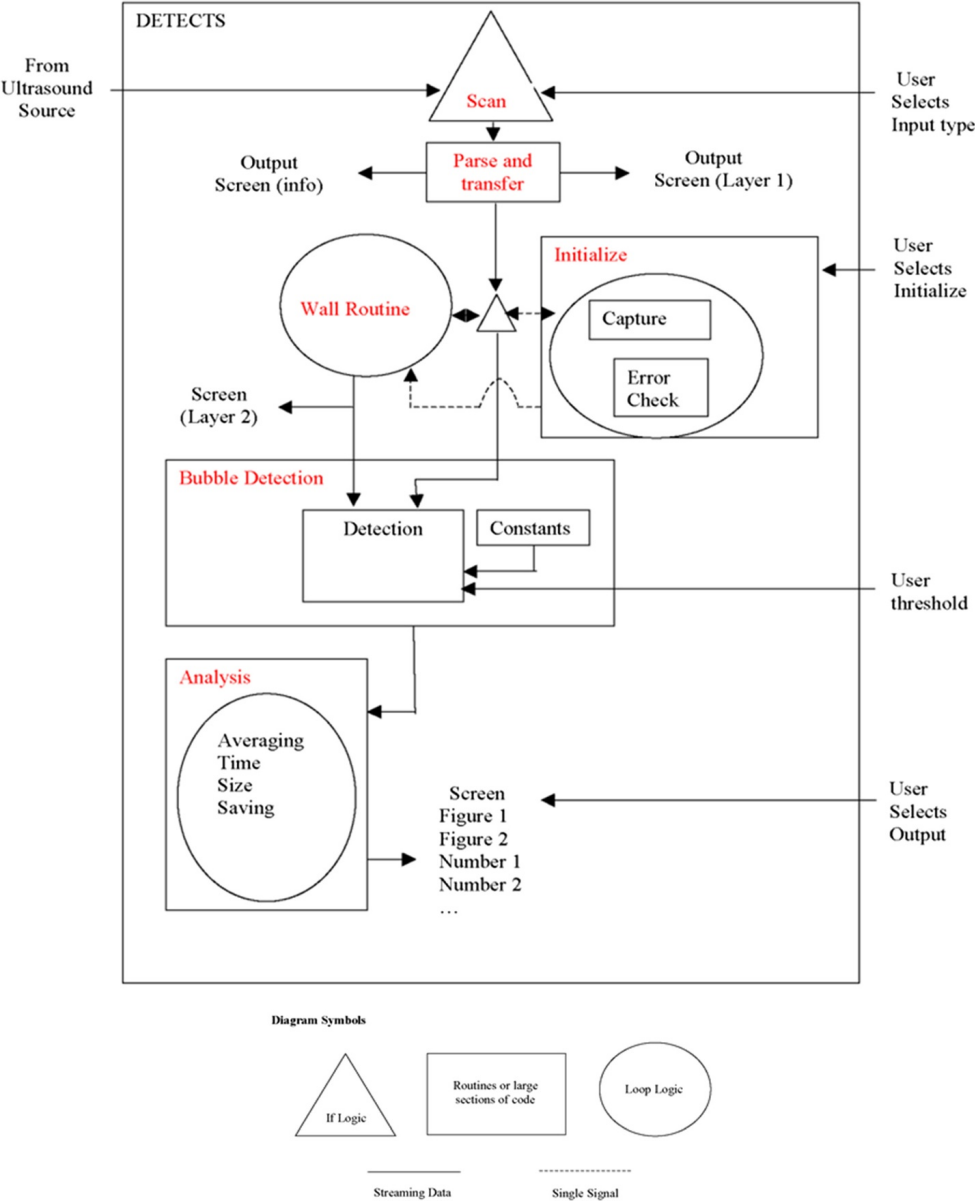
**DETECTS™ algorithm.** Block diagram of the DETECTS™ algorithm (U.S. Patent Application No. 12/289,918).

**Figure 2:**
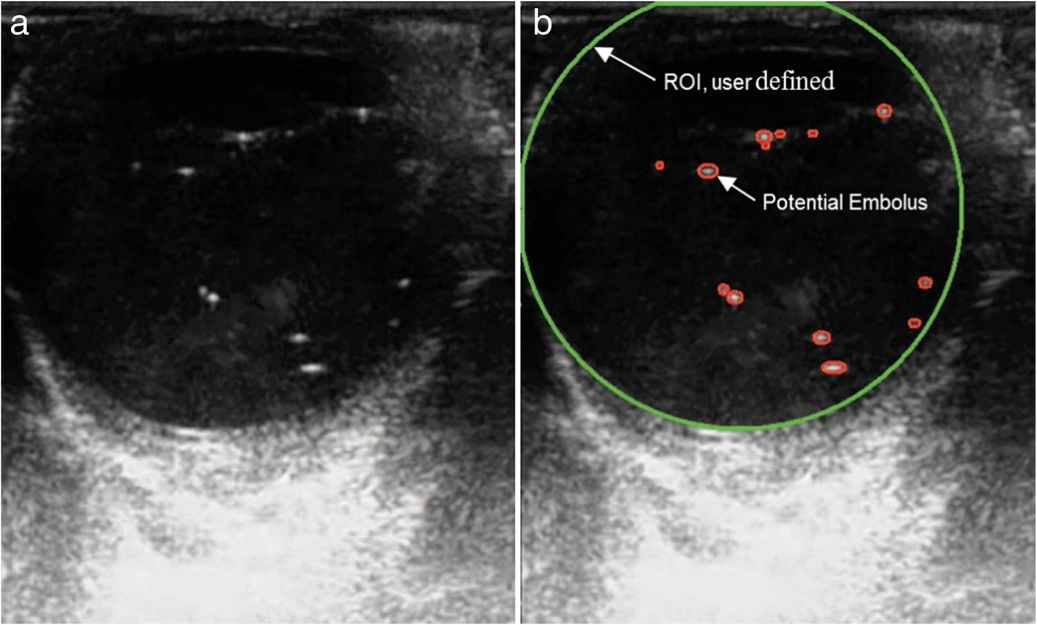
**TEE image of the ascending aorta. (a)** Original TEE image, and **(b)** DETECTS™ display, of the ascending aorta. In panel b, all potential emboli identified by DETECTS™ are circled in red, and the region of interest is circled in green in panel b.

The DETECTS™ algorithm was designed to be used in conjunction with TEE images of the ascending aorta or the chambers of the heart. Figure 1 shows a block diagram of the DETECTS™ algorithm, currently under patent review (U.S. Patent Application No. 12/289,918). When DETECTS™ initializes, the user defines a region of interest (ROI) such as the area bound by the aorta, or a heart chamber. The algorithm periodically updates the ROI using a wall gradient method to ensure accurate volume percentage calculations. DETECTS™ analyzes consecutive images from the TEE machine and displays modified TEE data to the user without interfering with normal TEE operation or visual outputs (Figure 2). From the two-dimensional (2D) TEE images, DETECTS™ measures the reflected acoustic signal from air bubbles to produce a 2D slice of the measurement volume. The intensity and size of the acoustic signal varies with the size of each air bubble being measured, as well as the input acoustic frequency, power, bubble shape, and number of bubbles in the vicinity (due to reflections and absorptions from other bubbles). The potential air emboli are checked with a set of rules to distinguish true embolus signals from false signals such as tissue signals. These rules include greyscale threshold, maximum and minimum aspect ratio, and size of the potential air emboli. Furthermore, DETECTS™ examines each potential signal for a continuous and smooth boundary. In this way, DETECTS™ identifies the presence of potential air emboli and provides a current and averaged embolus count, air volume measurement, size distribution, and three-dimensional (3D) reconstruction of the historical data from the ascending aorta or the chambers of the heart. The 3D reconstruction of the ultrasound data was designed to provide an easily interpreted visual feedback system for the operative team during the de-aeration process. Three-dimensional reconstruction provides a historical view of gaseous emboli that have passed by the measurement plane to emphasize clusters of gaseous emboli that have been released from the heart.

**Figure 3:**
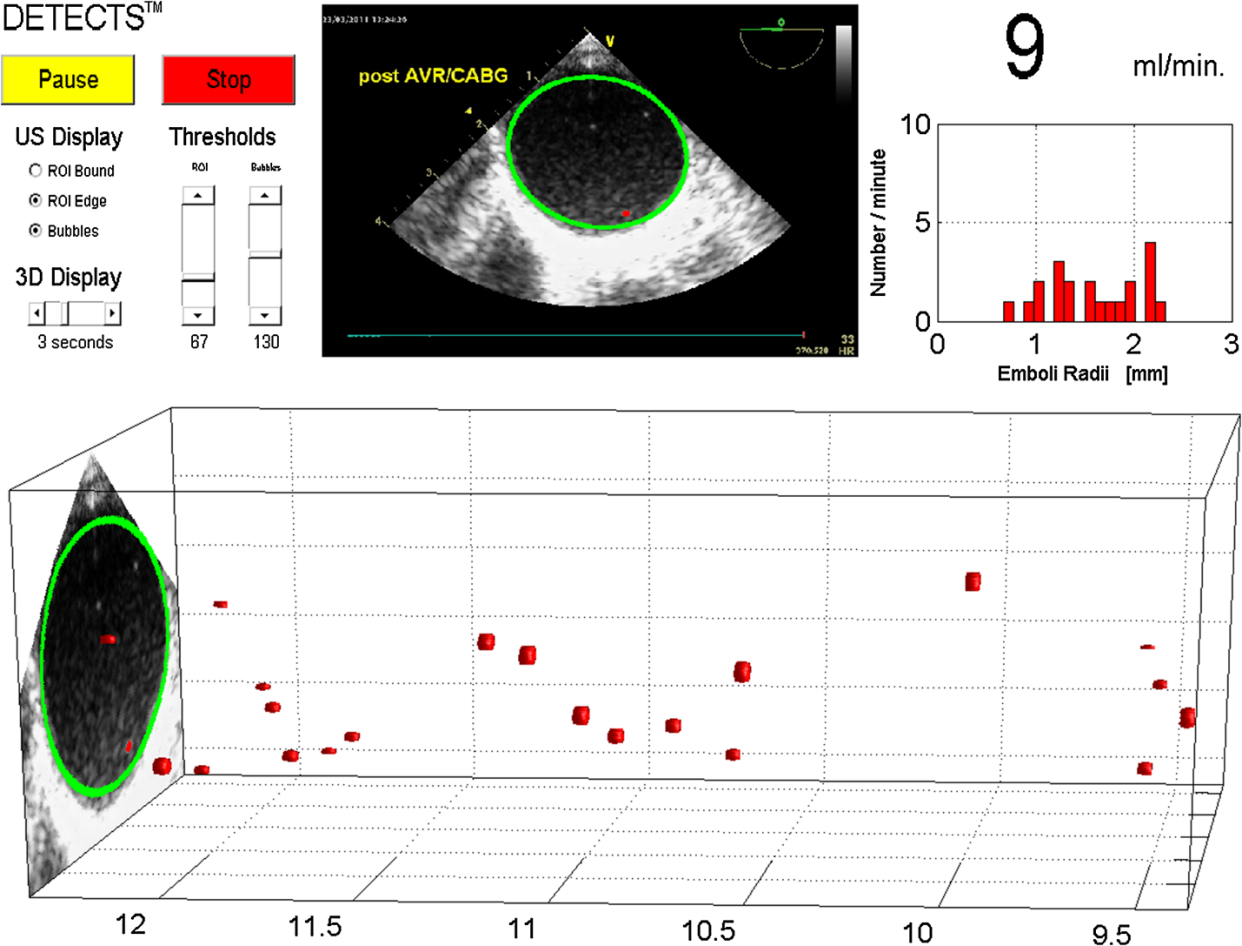
**DETECTS™ display.** Screenshot of the DETECTS™ algorithm displaying the original TEE image, average flow rate and size distribution of air emboli, and a three-dimensional reconstruction of the data.

The TEE images obtained during cardiac surgery using the DETECTS™ graphical user interface is shown in Figure 3 and Additional file 1. As depicted in Figure 3, the original TEE image is visible to the user along with the potential air emboli and a ROI marked. The user can adjust the program’s detection settings if needed. Offline testing of TEE images from cardiac procedures has shown exceptional program speed and accuracy (Additional file 1). Each ultrasound image frame is analyzed, and since the velocity of the flow is unknown, DETECTS™ counts each slice of a potential air embolus as it passes through the image plane. The 3D reconstruction connects the individual slices and counts the result as a single potential air embolus. The axes of the 3D reconstruction are x, y, and time, where the z-component (the location of the transducer) is assumed fixed. The x-y location of the bubble in the plane is calculated using the average of all the bubble slices that occurred as viewed in the ultrasound images. DETECTS™ links the individual occurrences of a bubble in consecutive frames and estimates the radius and location of the bubble. As time proceeds forward, the bubble is moved along the time axis maintaining its calculated x, y position. It is important to note that DETECTS™ does not show the full path of the bubbles visualized; rather, it provides an estimate of the volume of air passing through the plane of the TEE probe placed on the ascending aorta.

### *In vitro*validation of DETECTS™

**Figure 4:**
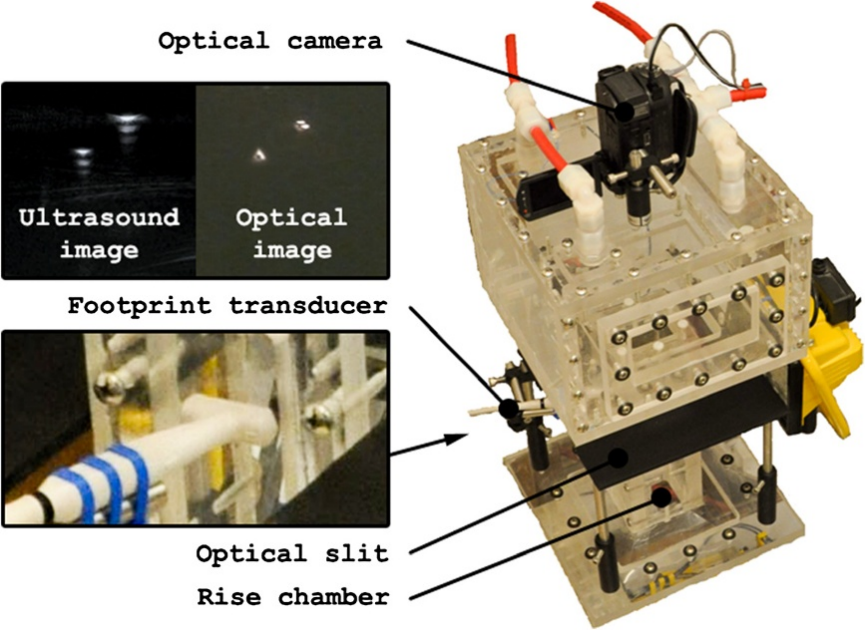
**Experimental setup for** ***in vitro*** **validation.** Experimental rig (right) highlighting optical and ultrasound images (top left) and footprint transducer attachment (bottom left).

We performed an *in vitro* experiment to compare the relationship between true bubble size (measured optically), and bubble size calculated by the DETECTS™ algorithm. To do this, we set up an experimental apparatus (Figure 4) that allowed air bubbles in tap water to be measured simultaneously using both DETECTS™ and a high-definition camera.

Our apparatus was comprised of a “bubbler” at the base of a clear Plexiglas™ rise chamber, through which air bubbles could rise in tap water. Air was delivered into the rise chamber through an air input using a pneumatic cylinder connected to a stepper motor. The air bubbles were produced at the ends of glass tubes connected to one of the air inputs. A portion of one wall of the rise chamber was replaced with a laminated plastic sheet. An optical measurement plane was created using two perpendicular 250 Watt halogen lamps that shone into the rise chamber through a narrow slit to produce a light sheet inside the middle of the rise chamber. A VF13-5SP footprint transducer was oriented in line with the optical slits on the side of the rise chamber, coupled to the laminated plastic sheet with ultrasonic gel to eliminate any air gaps. The rest of the validation apparatus was covered with black photographer’s background cloth.

DETECTS™ software installed on a Siemens Acuson/Antares ultrasound machine was used to quantify the number of potential emboli in consecutive images from the VF13-5SP footprint transducer. A High Definition Sony Handycam™ video camera was oriented to view facing down into the rise chamber (ie. normal to the plane of the light sheet). This video camera recorded light reflected from the air bubbles at 250 frames per second as the bubbles passed through the light sheet created inside the rise chamber.

Small amounts of air were introduced into the bubbler by incrementally moving the stepper motor, which forced air through the pneumatic cylinder. Air bubbles with a radius ranging from 0.1 to 1.5 mm produced at the ends of the glass tubes inside the bubbler rose through the rise chamber, and travelled through the measurement plane of the optical and DETECTS™ systems for simultaneous detection by the cameras and the ultrasound transducer. The Matlab (R2009) imaging toolbox was used to analyze the optical frames. A computer program was created to automatically process each of the optical frames. This included thresholding the images using a basic greyscale threshold and then measuring the number and size of the bubbles in the frame; the result produced centre of geometrics and size estimations of the bubble slices in the optical images. A 3D reconstruction similar to that used for DETECTS™ was used to link the individual bubble slices together and estimate the volume of the bubble. The bubbles captured from the ultrasound were analyzed with the DETECTS™ algorithm directly without any modification. The data from the two imaging (optical and ultrasound) techniques were then compared in terms of the number of bubbles, the location of the bubbles, and the bubble radii. Correlation of the optical and ultrasound data used a least squares technique which minimized the total error of three specific parameters (the position, time, and radius differences).

**Figure 5:**
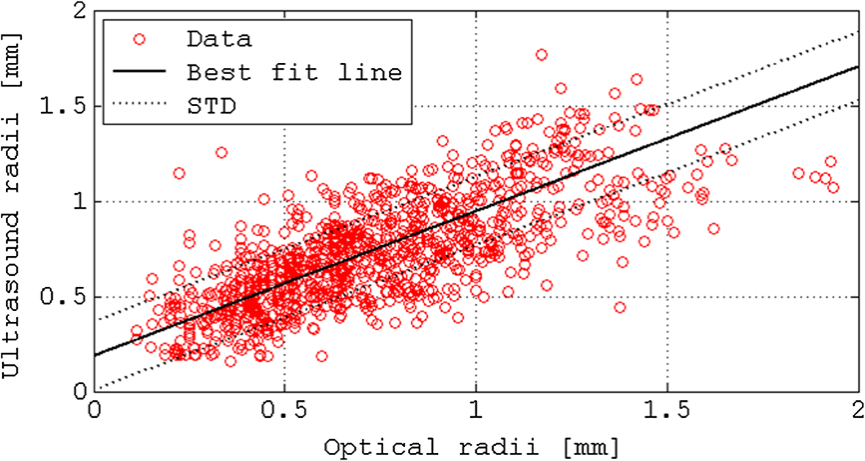
**Bubble radius correlation.** Correlation of the bubble radii obtained from ultrasound and optical data. STD = standard deviation.

**Figure 6:**
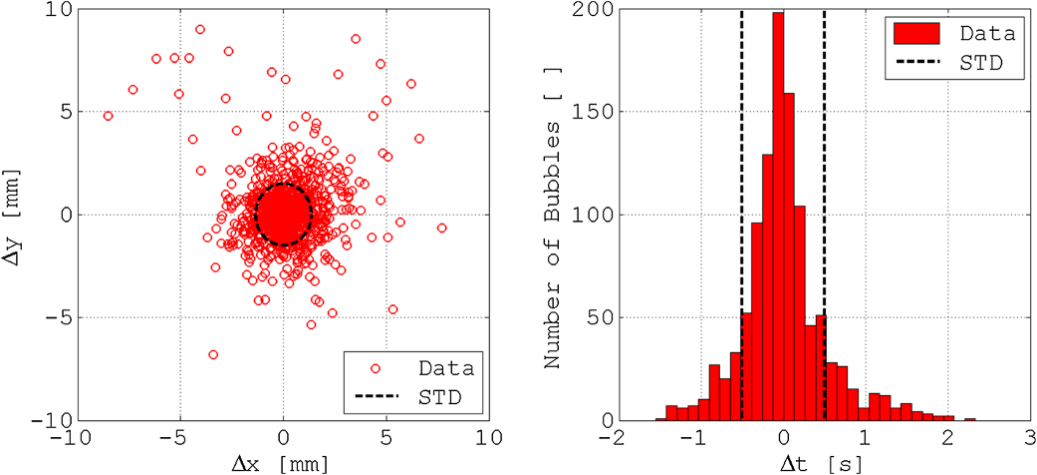
**Bubble centre of geometry and time difference correlation.** Difference in centre of geometry correlation (left) and time difference correlation (right) of ultrasound and optical data. STD = standard deviation.

Bubble radius measurements obtained with each imaging technique were correlated (r = 0.76), with 907 of 1030 bubbles correlated between the optical and ultrasound measurements (Figure 5). Occasional challenges in 3D reconstructions resulted in 123 bubbles being uncorrelated. In these instances, the ultrasound measurements produced the unexpected phenomenon of phantom bubbles for each true bubble due to the low acoustic impedance between the transducer and the bubbles. Evidence of these phantom bubbles can be seen in the upper left inset of Figure 4. In the ultrasound image, the air bubbles have two or three distinct dots, whereas air bubbles have only one dot in the optical image. Although the 3D reconstruction was used to correct this, noise was still present in the final results. However, this issue should not be expected *in vivo* since the acoustic impedance is significantly higher due to the endothelium of the aorta. The centre of geometry and the time difference of the optical and ultrasound data are shown in Figure 6. One standard deviation for the planar position difference and the time difference was calculated to be 1.4 mm and 0.4 seconds, respectively. Due to the characteristics of air bubbles in water, each air embolus had a distinct wobble, which resulted in the relatively small 1.4 mm and 0.4 second error estimates. Both of these values fall within the 3D reconstruction noise.

The thresholds to extract each bubble from the ultrasound and optical data were determined. The mechanical index and gain on the ultrasound machine were adjusted to test the sensitivity of the bubble threshold value calculated using DETECTS™ versus the mechanical index and ultrasound image gain. The bubble threshold value was more influenced by the mechanical index than by the image gain, but both results indicated that DETECTS™ is able to extract the bubble threshold value for an extreme range of ultrasound inputs.

## Conclusion

Cardiac surgery is commonly performed in the elderly population, which is particularly susceptible to post-operative neurologic dysfunction [11, 12]. Since neurological dysfunction is thought to be in part caused by cerebral air microemboli [4], efforts are taken by operative teams to reduce microembolism associated with heart surgery [7]. Despite these efforts, air bubbles are frequently observed with TEE after a cardiac surgery patient is separated from CPB [10]. Although TEE alone is a useful tool for determining the presence of potential emboli in the blood postoperatively, and it is commonly used to qualitatively estimate air trapped in the heart, it is unable to accurately quantify the number of bubbles in the heart.

Our new system incorporating the DETECTS™ software algorithm conveniently integrates the use of TEE technology to provide an accurate estimate of the number and size of air bubbles in the heart. Qualitative and quantitative analysis provided promising results from the DETECTS™ algorithm compared to optical measurements. The shape and intensity of the reflected acoustic signal was determined to uniquely characterize the true bubble radius; individual air bubbles could be reliably distinguished from each other. Although there was some noise in the bubble 3D reconstruction due to phantom bubbles, DETECTS™ still showed a strong correlation between optical and ultrasound measurements, with minimal centre of geometry and time differences between the measurement techniques.

By enabling the operative team to visualize the number and size of air emboli in the cardiac chambers and/or aorta in real time, it is our hope that they will adjust their de-airing techniques to further reduce the amount of air trapped in the heart. Ultimately, we believe this will lead to improved de-airing techniques and, in turn, reduced rates of neurological dysfunction in cardiac surgery patients. In the future, DETECTS™ may also prove useful for the evaluation of other cardiac conditions associated with microembolism, and for the detection of bubbles and potentially solid particles in other flow systems.

### Future directions

Future work in the development of DETECTS™ will ideally involve assessment of the software performance using blood products. It was necessary in the current study to use water instead of blood to allow for the use of optical measures to validate DETECTS™ by comparing optical and ultrasound output. Future studies should provide data on the capacity of DETECTS™ to estimate the volume of air in blood. Furthermore, once permitted for *in vivo* use, the clusters of gaseous emboli visualized by DETECTS™ during cardiac surgery in real time could be correlated to the surgeon’s maneuvers to gauge the most effective technique to remove air from the heart.

### Availability and requirements

Project Name: Detection of Emboli using Transesophageal Echocardiography for Counting, Total volume, and Size estimation

Project Homepage: N/A

Operating System(s): Windows XP Eng mode (on ACUSON Antares Ultrasound Machine)

Programming Language: C++

Other Requirements: N/A

License: There is no license for this product; however, it is currently under review for a patent

Any restrictions to use by non-academics: Patent pending

## Authors’ information

FS is a PhD candidate in the Department of Mechanical and Materials Engineering at Queen’s University, Canada. AP is a Professor and Queen’s Research Chair in Fluid Dynamics and Multi-scale Phenomena in the Department of Mechanical and Materials Engineering at Queen’s University, Canada. CGB has a MSc in Mechanical Engineering from Queen’s University, Canada, and is currently a resident in Anatomical Pathology at the University of Ottawa in the Department of Pathology and Laboratory Medicine, Canada. MU is an Associate Professor in the Department of Mechanical Engineering and Engineering Science at University of North Carolina Charlotte, USA, and is the Director of NC Motorsports and Automotive Research Center, USA. AH is an Associate Professor, Chair of the Cardiac Division, and Deputy Head of the Department of Surgery at Queen’s University, Canada, and is an attending cardiac surgeon at the Kingston General Hospital, Canada. RCT is an Assistant Professor in the Department of Anesthesiology & Perioperative Medicine at Queen’s University, Canada, and is an attending anesthesiologist at the Kingston General Hospital, Canada. JBT holds a PhD in Psychology, Neuroscience & Behaviour, and is a Clinical Research Associate in the Department of Anesthesiology & Perioperative Medicine at Queen’s University, Canada. BM is an Adjunct Professor in the Department of Anesthesiology & Perioperative Medicine at Queen’s University, Canada, Professor Emeritus at Queen’s University, Canada, and a former attending anesthesiologist at the Kingston General Hospital, Canada.

## Electronic supplementary material

Additional file 1: **DETECTS™ display of TEE images obtained during cardiac surgery.** Live feed of DETECTS™ display during its use on a cardiac surgery patient. The TEE image of the aorta and three-dimensional reconstruction of potential air emboli, as well as the radius of each potential embolus, are shown. (ZIP 17 MB)
